# Solution NMR of Battery Electrolytes: Assessing and Mitigating Spectral Broadening Caused by Transition Metal Dissolution

**DOI:** 10.1021/acs.jpcc.2c08274

**Published:** 2023-02-28

**Authors:** Jennifer P. Allen, Clare P. Grey

**Affiliations:** † Yusuf Hamied Department of Chemistry, University of Cambridge, Lensfield Road, Cambridge, CB2 1EW, Cambridge, United Kingdom; ‡ The Faraday Institution, Quad One, Harwell Science and Innovation Campus, Didcot OX11 0RA, United Kingdom

## Abstract

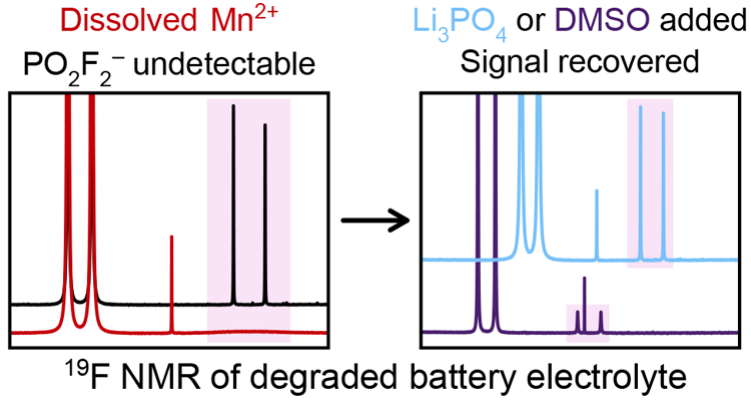

NMR spectroscopy is a powerful tool that is commonly used to assess the degradation of lithium-ion battery electrolyte solutions. However, dissolution of paramagnetic Ni^2+^ and Mn^2+^ ions from cathode materials may affect the NMR spectra of the electrolyte solution, with the unpaired electron spins in these paramagnetic solutes inducing rapid nuclear relaxation and spectral broadening (and often peak shifts). This work establishes how dissolved Ni^2+^ and Mn^2+^ in LiPF_6_ electrolyte solutions affect ^1^H, ^19^F, and ^31^P NMR spectra of pristine and degraded electrolyte solutions, including whether the peaks from degradation species are at risk of being lost and whether the spectral broadening can be mitigated. Mn^2+^ is shown to cause far greater peak broadening than Ni^2+^, with the effect of Mn^2+^ observable at just 10 μM. Generally, ^19^F peaks from PF_6_^–^ degradation species are most affected by the presence of the paramagnetic metals, followed by ^31^P and ^1^H peaks. Surprisingly, when NMR solvents are added to acquire the spectra, the degree of broadening is heavily solvent-dependent, following the trend of solvent donor number (increased broadening with lower solvent donicity). Severe spectral broadening is shown to occur whether Mn is introduced via the salt Mn(TFSI)_2_ or is dissolved from LiMn_2_O_4_. We show that the weak ^19^F and ^31^P peaks in spectra of electrolyte samples containing micromolar levels of dissolved Mn^2+^ are broadened to an extent that they are no longer visible, but this broadening can be minimized by diluting electrolyte samples with a suitably coordinating NMR solvent. Li_3_PO_4_ addition to the sample is also shown to return ^19^F and ^31^P spectral resolution by precipitating Mn^2+^ out of electrolyte samples, although this method consumes any HF in the electrolyte solution.

## Introduction

Lithium-ion cells degrade by numerous chemical and mechanical processes occurring at the cathode and anode surfaces and within the electrolyte.^1,2^ One pathway by which cells degrade is transition metal dissolution, whereby the metals in metal-oxide cathodes leach out of the material and into the electrolyte solution.^3−8^ While dissolution of Mn, Ni, Co, and Fe are all harmful, the severity of dissolution-induced capacity losses appears worse for Mn than for other transition metals.^9−15^ Hence, transition metal dissolution from the cathode materials LiMn_2_O_4_ (LMO),^16−25^ LiMn_*x*_Ni_2–*x*_O_4_ (LNMO),^26−29^ and LiNi_*x*_Mn_*y*_Co_1–*x*–*y*_O_2_ (NMC)^12,14,30−36^ has been widely studied. In cells, dissolved metals generally migrate to and accumulate at the anode;^3,5,6,8^ however, some metal remains in solution (particularly for cells stored at high voltages or temperatures and for symmetric cells, as well as in ex situ leaching experiments where cathode powders are stored in electrolyte solutions). Analyses vary widely due to different materials, electrolyte volumes, temperatures, timeframes, and electrochemical conditions, but a survey of the literature reveals that Ni and Mn from stoichiometric or lithium-rich LMO, LNMO, and NMC have been measured in LiPF_6_ solutions on the order of μM–mM.^10,17,30,31,34,35,37−59^ Dissolution/extraction of surface films, separators, and electrodes into reasonable volumes also yields values in the μM–mM range.^35,60−70^

Another route of capacity loss in lithium-ion cells is the chemical or electrochemical degradation of the electrolyte solution.^1,2^ These electrolyte degradation products can be identified and quantified at low concentrations through rapid, nondestructive solution NMR experiments.^71^ Soluble degradation species within electrolyte solutions are well-suited for study by NMR due to the presence of abundant NMR active nuclides including ^1^H (in electrolyte solvents), ^19^F (in many Li salts), and ^31^P (in LiPF_6_, the most commonly used Li salt). Additional nuclides such as ^7^Li, ^17^O, and ^13^C are present, but may be less useful due to narrow chemical shift windows (^7^Li) and low natural abundances and thus sensitivities (^17^O and ^13^C).

NMR investigation of degradation species in lithium-ion battery electrolyte solutions is typically performed ex situ (i.e., samples are from model experiments or from post-mortem cells). These experiments are generally performed by (i) mixing electrolyte solutions with (or extracting them into) deuterated solvents including dimethyl sulfoxide-*d*_6_ (DMSO),^72−77^ CD_3_CN,^78−82^ CDCl_3_,^83−86^ tetrahydrofuran-*d*_8_,^87−89^ and acetone-*d*_6_;^90^ or (ii) using electrolyte solutions neat^69,91−101^ or diluted with/extracted into dimethyl carbonate^102−104^ or ethyl methyl carbonate (EMC).^105^ In case (ii), deuterated solvents are incorporated into, but are physically separated from, the sample (e.g., via use of a solvent capillary). NMR analysis has also been performed on dissolved solids, such as precipitates formed in electrolyte solutions and surface films on electrodes, with solvents including D_2_O,^106−115^ DMSO-*d*_6_,^116−119^ C_6_D_6_,^91^ acetone-*d*_6_,^114^ DCl in D_2_O,^118^ and EMC,^120^ while CD_3_CN has been found not to dissolve solid electrolyte interphase (SEI) components.^81^ In some cases, researchers rinse cell parts with solvents to extract the remaining electrolyte solution, but others do the same in order to dissolve surface films, so the difference between solution NMR of solutions and solution NMR of dissolved solids is not always clear. Sample preparation is relevant to the incorporation of transition metals into the NMR sample; for instance, with a flooded cell, removing the electrolyte solution and diluting it with solvent, soaking the separator in solvent, or soaking electrodes in solvent may result in samples with similar degradation species but different transition metal concentrations.

Given the prominence of solution NMR to study electrolyte degradation and the existence of metal dissolution as a degradation mechanism, it is therefore plausible that NMR samples may contain dissolved transition metals. This is significant because the dissolved Ni and Mn ions that have been observed in LiPF_6_ electrolyte solutions (i.e., the oxidation states relevant to battery chemistry) are Ni^2+^, Mn^2+^, and Mn^3+^,^17,18,23,32,34,37,39,48,52,60,62,63,121^ which are paramagnetic. Furthermore, paramagnetic Mn^0^, Mn^2+^, Mn^3+^, Mn^4+^, and Ni^2+^ have been observed in the SEI,^14,21,27,32,122−127^ such that dissolution of surface films for analysis may also lead to paramagnetic contamination of NMR samples.

The presence of paramagnetic species in NMR samples leads to enhanced nuclear relaxation, whereby unpaired electron spins on the metals cause fluctuating magnetic fields that induce nuclear spin-flip transitions.^128,129^ As a result, nuclei in samples containing paramagnetic species undergo more efficient relaxation, with faster longitudinal (*R*_1_) and transverse (*R*_2_) relaxation rates.^130^ The peak widths of resonances in NMR spectra of paramagnetic solutions are controlled by factors including the metal concentration, its number of unpaired electrons, its electron spin relaxation time, the concentration of any species coordinating the metal, the distance of those species from the metal, and any chemical exchange within the metal coordination sphere (addressed further in the Discussion).^130−134^ Thus, the dissolved transition metals in battery solution NMR samples may induce rapid nuclear relaxation and spectral broadening that could obfuscate the observation of degradation species, particularly those present at low concentrations.

This work aims to establish how the presence of Ni^2+^ and Mn^2+^ in LiPF_6_ electrolytes affects NMR studies of the solutions, and whether such effects can be mitigated. This is explored in pristine electrolytes, degraded electrolytes, and in electrolytes stored with LiMn_2_O_4_; sample dilution and metal precipitation are explored as two possible methods to improve spectral resolution. It is shown that while dissolved transition metals minimally affect the peaks from the majority electrolyte components found in the pristine solutions, the small peaks of degradation species can be significantly broadened. Broadening is far more severe in the presence of Mn^2+^ than Ni^2+^, and PF_6_^–^ degradation species appear most strongly affected by metal dissolution. In samples diluted with NMR solvents, peak broadening is mitigated best with DMSO; broadening is also mitigated via Li_3_PO_4_ addition and subsequent metal precipitation, at the cost of removing HF from solution. This work shows that Mn^2+^ dissolved from battery cathodes can substantially affect the identification of degradation products by NMR, and we propose that high-donor number solvents be preferentially used for NMR of electrolyte solutions so as to obtain more representative spectra of degradation species.

## Methods

### Sample Preparation

All samples were prepared in an argon glovebox and all NMR tubes were sealed under argon. A solution of 1 M LiPF_6_ in 3:7 ethylene carbonate/ethyl methyl carbonate (EC/EMC, v/v) was sourced premixed (soulbrain R&D, H_2_O < 20 ppm by Karl Fischer titration). The bis(trifluoromethane)sulfonimide (TFSI) salts Mn(TFSI)_2_ (Solvionic, 99.5%) and Ni(TFSI)_2_ (Alfa Aesar, 97+%) were used as the sources of paramagnetic metals and were added in concentrations of 0.01, 0.05, 0.1, 0.5, 1.0, and 5.0 mM to the LiPF_6_ solutions. For the degraded electrolyte solution, 30 mL of diamagnetic electrolyte was sealed in a plastic centrifuge tube with 150 μL of deionized water and stored at 60 °C for 2 weeks, after which transition metals were added. To examine metal precipitation as a route to restore spectral resolution, electrolyte samples that had been heat- and water-degraded (12 μL of water in 12 mL of electrolyte solution, stored at 60 °C) containing 1 mM Mn(TFSI)_2_ were shaken with ∼10 mg of Li_3_PO_4_ (Sigma-Aldrich).

For all experiments including deuterated solvents, CD_3_CN (Fluorochem), CD_3_OD (Sigma-Aldrich), or DMSO-*d*_6_ (Sigma-Aldrich) were mixed in a 9:1 volume ratio with electrolyte samples. Methanol has been observed in NMR studies of degraded electrolyte solutions,^74,78^ so it is generally not advisible to use MeOD as a solvent when studying electrolyte solutions; it is used herein only as an additional point of comparison with the more commonly used solvents DMSO-*d*_6_ and CD_3_CN.

### LiMn_2_O_4_ Storage

To compare Mn(TFSI)_2_ to Mn dissolved from cathodes, 3 g of LiMn_2_O_4_ was mixed with 7 mL of electrolyte solution. The sample, stored under argon, was heated at 60 °C for 77 days, then was centrifuged to remove the electrode powder. NMR spectra of the undiluted electrolyte solution and samples diluted 10× with CD_3_CN or DMSO-*d*_6_ were measured. The Mn concentration in the electrolyte solution was determined by inductively coupled plasma optical emission spectroscopy (ICP-OES). Triplicate samples were prepared by dilution with nitric acid (trace metal grade). ICP-OES measurements were performed on an iCAP 7400 Duo ICP-OES Analyzer (Thermo Fisher Scientific).

### Solution NMR

^1^H, ^19^F, and ^31^P spectra were acquired on a Bruker 500 MHz Avance III HD spectrometer using a broadband observe (BBO) probe. All undiluted electrolyte samples contained a capillary of C_6_D_6_ for field locking and referencing (i.e., no deuterated solvents were in contact with the electrolyte). The samples diluted with deuterated solvents (DMSO-*d*_6_, CD_3_CN, or MeOD) were locked and referenced to whichever solvent was mixed with the electrolyte; these samples did not contain solvent capillaries. The purpose of this work is to study spectral broadening, not peak positions; hence, undiluted samples containing paramagnetic solutes were referenced to diamagnetic shifts to eliminate the bulk magnetic susceptibility shift. All NMR experiments used a 30° pulse, with recycle delays of 1 s for ^1^H and ^19^F experiments and 2 s for ^31^P experiments.

## Results

### Metal Addition to Pristine Electrolyte Solutions

**Figure 1:**
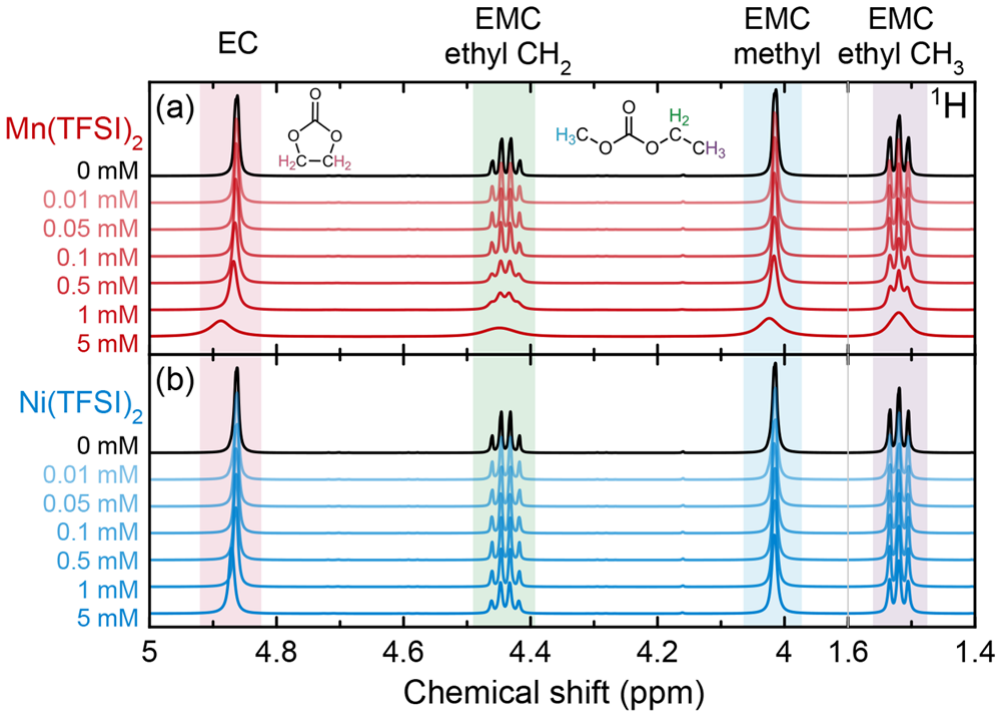
^1^H NMR of pristine electrolyte solutions (1 M LiPF_6_ in 3:7 EC/EMC) containing Mn(TFSI)_2_ or Ni(TFSI)_2_. The diamagnetic spectrum is referenced to a C_6_D_6_ capillary; paramagnetic spectra are referenced to the EMC ethyl CH_3_ resonance at 1.52 ppm (eliminating the bulk magnetic susceptibility shift). Only the 1.4–1.6 ppm and 3.9–5 ppm ranges are shown, where the EC and EMC resonances are found. Peaks at 4.86 ppm (shaded in pink), 4.44 ppm (shaded in green), 4.02 ppm (shaded in blue), and 1.52 ppm (shaded in purple) indicate the EC, EMC ethyl CH_2_, EMC methyl, and EMC ethyl CH_3_ environments, respectively.

Figure 1 shows the ^1^H NMR of pristine electrolyte solutions of 1 M LiPF_6_ in 3:7 EC/EMC (v/v) containing 0–5 mM of Mn(TFSI)_2_ or Ni(TFSI)_2_. The paramagnetic spectra were referenced to the diamagnetic spectrum to highlight the peak broadening, as paramagnetic solutes can also cause shifts that obfuscate differences in peak widths. This peak shifting is largely caused by bulk magnetic susceptibility (BMS) shifts that affects all peaks equally.^128,135^ Any shift mechanisms that result in site-specific shifts and affect the peaks differentially, that is, via contact or pseudocontact mechanisms, should still be visible via this approach. Notably, the EC peak is affected by a small hyperfine shift due to transition metal coordination,^136^ which is not removed by accounting for the BMS shift; thus, the EC shifts at 5 mM Mn^2+^ and 5 mM Ni^2+^ are not aligned with the EC shifts at lower metal concentrations. ^1^H spectra were referenced to the EMC ethyl CH_3_ resonance at 1.52 ppm, ^19^F spectra were referenced to the PF_6_^–^ resonance at −74.40 ppm, and ^31^P spectra were referenced to the PF_6_^–^ resonance at −144.34 ppm.

The broadening caused by the addition of Mn(TFSI)_2_ is far more severe than the broadening caused by Ni(TFSI)_2_. In particular, after the addition of 5 mM Mn(TFSI)_2_, a complete loss of resolution of the ^1^H–^1^H *J*-coupling occurred, with the EMC quartet at 4.44 ppm and the triplet at 1.52 ppm appearing as singlets. By contrast, the loss of resolution at 5 mM Ni(TFSI)_2_ appears comparable to the loss of resolution observed at 0.05–0.1 mM Mn(TFSI)_2_.

The solvent ratio gives expected peak integrals for the EC peak, EMC ethyl CH_2_ peak, EMC methyl peak, and EMC ethyl CH_3_ peak of 18.0:13.5:20.3:20.3, respectively. The weakest peak is the EMC ethyl CH_2_, with its 1:3:3:1 quartet at 4.44 ppm caused by ^1^H–^1^H *J*-coupling; this appears as a broad resonance at the highest Mn concentration. Yet, even with the most severe paramagnetic contamination at 5 mM Mn(TFSI)_2_, the EMC ethyl CH_2_ resonance and all other peaks are clearly detectable.

**Figure 2:**
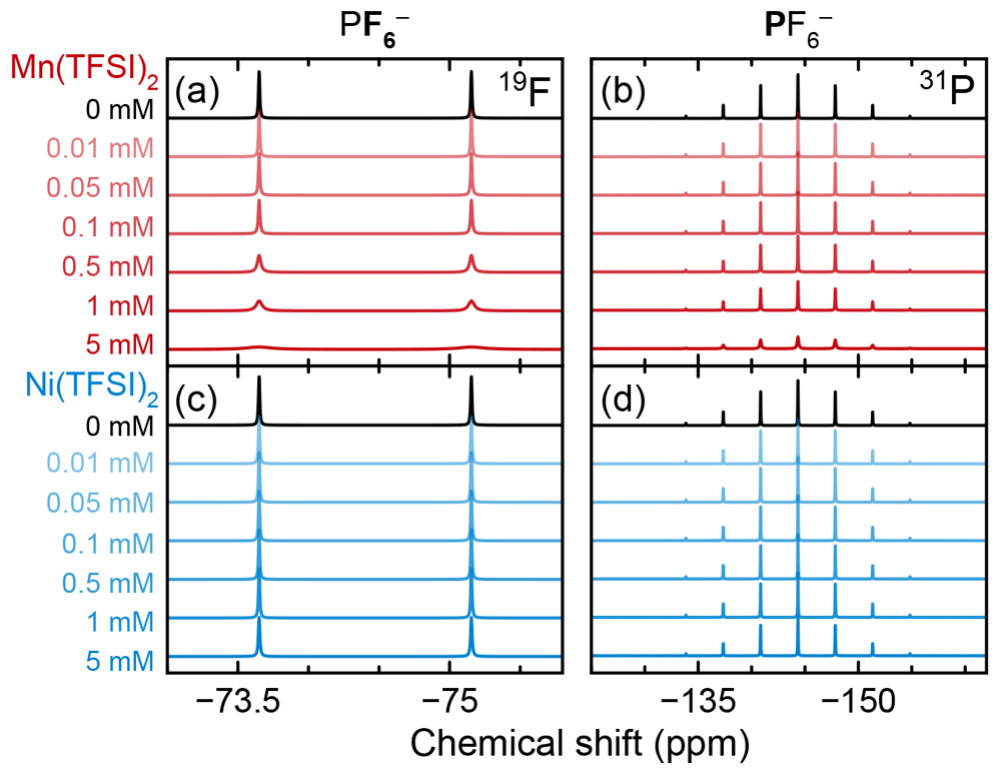
(a, c) ^19^F and (b, d) ^31^P NMR of pristine electrolyte solutions containing Mn(TFSI)_2_ or Ni(TFSI)_2_. The spectral ranges containing resonances from the PF_6_^–^ anions are shown (the ^19^F TFSI^–^ resonance appears at −80.2 ppm and is not shown here). Paramagnetic spectra are referenced to the PF_6_^–^ resonances at −74.40 ppm (^19^F) and −144.34 ppm (^31^P) for ease of comparison (eliminating the bulk magnetic susceptibility shift).

Figure 2 shows the ^19^F and ^31^P NMR of the same pristine electrolyte solutions, where the peaks arise from the PF_6_^–^ anion. In all cases, the coupling is not obscured and the individual peaks of the *J*-multiplets remain detectable. The ^1^H–^1^H coupling is on the order of 7 Hz, while the ^31^P–^19^F coupling is on the order of 700 Hz, so the preservation of ^31^P–^19^F coupling is unsurprising. As with ^1^H NMR (Figure 1), the effect of Mn(TFSI)_2_ on peak width is far more pronounced than the effect of Ni(TFSI)_2_.

### Degraded Electrolyte Solutions

**Figure 3:**
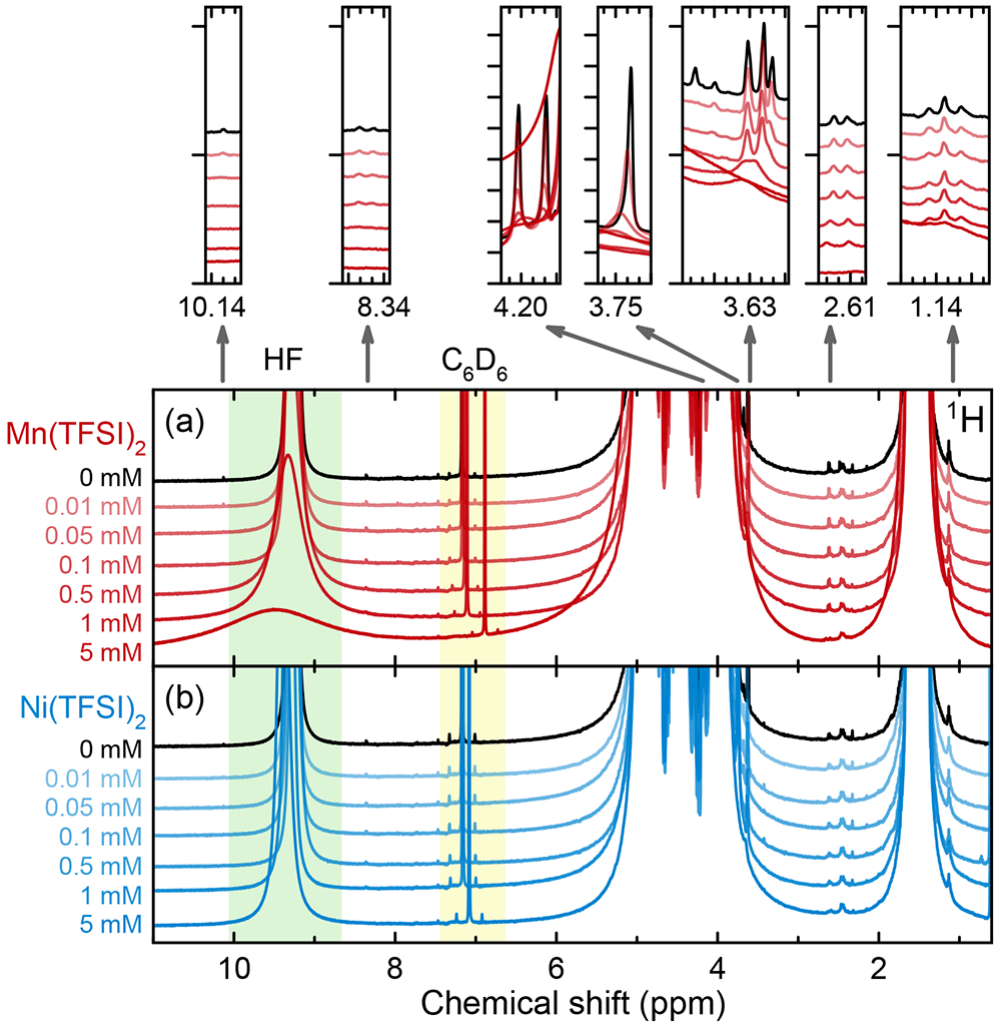
^1^H NMR of degraded electrolytes containing Mn(TFSI)_2_ or Ni(TFSI)_2_. Diamagnetic spectrum is referenced to a C_6_D_6_ capillary; paramagnetic spectra are referenced to the EMC ethyl CH_3_ resonance at 1.52 ppm for ease of comparison (eliminating the bulk magnetic susceptibility shift). Green shaded area (∼9.3 ppm) indicates HF; yellow shaded area (∼7.2 ppm) indicates residual hydrogens on C_6_D_6_. Expanded spectra show broadening of select degradation peaks; *x*-axis minor tick marks indicate 0.01 ppm and *y* axes are scaled consistently, where each increment is 1 arbitrary unit.

Figure 3 shows the ^1^H NMR spectra of 1 M LiPF_6_ in 3:7 EC/EMC solutions that were degraded via water addition and 60 °C storage, after which 0–5 mM Mn(TFSI)_2_ or Ni(TFSI)_2_ was added.

The large HF peak at ∼9.3 ppm (shaded in green) is caused by PF_6_^–^ hydrolysis, from the added water. Since the degradation peaks are of interest here, the spectra are magnified and the solvent peaks are off-scale. Paramagnetic spectra are still referenced to diamagnetic EMC to remove the BMS shift, as was done for pristine solutions; hence, the C_6_D_6_ residual peak at ∼7.2 ppm (shaded in yellow) appears to shift with addition of the metals, as the C_6_D_6_ inside the capillary is not affected by the BMS shift. New peaks from alkyl environments are visible at ∼1–3 ppm, hydroxyl and alkoxide environments (including alkoxide ligands on fluorophosphate species) at ∼3–5 ppm, and aldehyde and acid species at ∼8–11 ppm. A more detailed analysis of the spectra of degradation products can be found elsewhere,^91−93,96^ noting that peak positions reported in *d*-solvents may differ from those reported in undiluted electrolyte solutions. The degree of peak broadening is not consistent for all degradation species and, of note, some peaks appear more susceptible to broadening than others.

**Figure 4:**
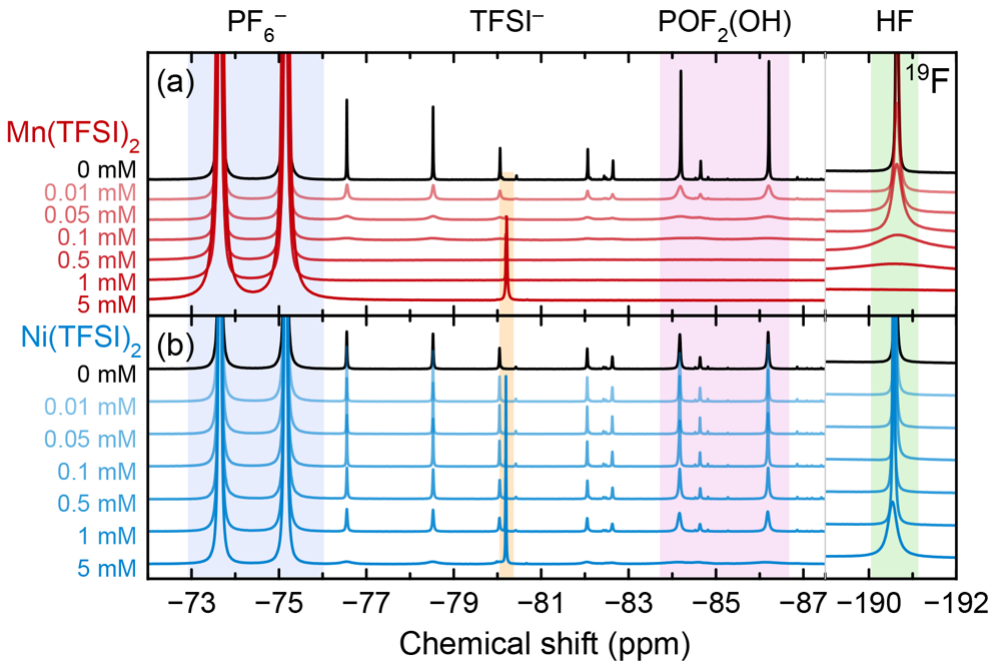
^19^F NMR of degraded electrolytes containing Mn(TFSI)_2_ or Ni(TFSI)_2_. Paramagnetic spectra are referenced to the PF_6_^–^ resonance at −74.40 ppm (shaded blue) for ease of comparison (eliminating the bulk magnetic susceptibility shift). Regions shaded in orange (−80.2 ppm), pink (−85.2 ppm), and green (−190.6 ppm) indicate TFSI^–^, POF_2_(OH), and HF, respectively.

Figure 4 shows the ^19^F NMR of the same electrolyte solutions degraded by heat and water. Here, major degradation species, other than HF, are all fluorophosphates, where the doublets (*J* = 929–950 Hz) arise from P–F coupling. For water-degraded samples, we assign the doublet at −85.2 ppm to POF_2_(OH) rather than PO_2_F_2_^–^ due to the high acid (HF) content. With only 0.01 mM Mn(TFSI)_2_, the ^19^F peaks are broadened dramatically, and the smallest peaks are almost undetectable; at 0.05 mM, the small peaks have disappeared, and by 0.5 mM Mn(TFSI)_2_, startlingly, it appears as though there are no degradation species in the electrolyte at all. The large HF peak at −190.6 ppm is no longer apparent after the addition of 5 mM Mn(TFSI)_2_. Again, the broadening effect is less severe with Ni(TFSI)_2_ than with Mn(TFSI)_2_, but after the addition of 5 mM Ni(TFSI)_2_, many of the smaller degradation peaks (from various fluorophosphates)^91−93,96^ are undetectable. In all cases, the PF_6_^–^ resonance at −74.40 ppm remains apparent and does not appear to undergo broadening as significant as that of the degradation peaks.

**Figure 5:**
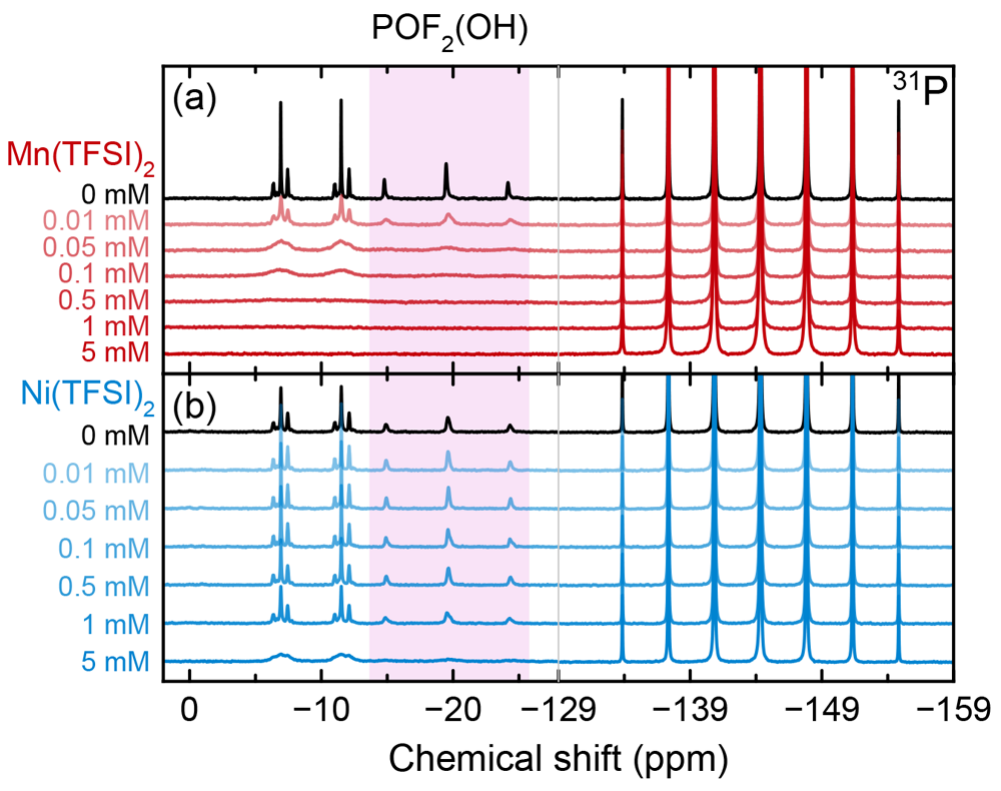
^31^P NMR of degraded electrolytes containing Mn(TFSI)_2_ or Ni(TFSI)_2_. Paramagnetic spectra are referenced to the PF_6_^–^ septet at −144.34 ppm for ease of comparison (eliminating the bulk magnetic susceptibility shift). The triplet at −19.6 ppm arises from POF_2_(OH) (shaded in pink).

Figure 5 shows the ^31^P NMR spectra of the same degraded electrolyte solutions analyzed in Figures 3 and 4. The triplet at −19.6 ppm (*J*_P–F_ = 950 Hz) is assigned to POF_2_(OH), while the other three major resonances are doublets (which appear at a distance almost as one doublet of triplets; d, *J*_P–F_ = 929 Hz; dt, *J*_P–F_ = 945 Hz, *J*_P–H_ = 8 Hz; dq, *J*_P–F_ = 945 Hz, *J*_P–H_ = 11 Hz). This is consistent with the ^19^F NMR and suggests degradation species of the form POF(OR_1_)(OR_2_), where R_1_ and R_2_ = H (from water), Me (from EMC), or Et (from EMC). As observed in Figure 2, the effect of the transition metals on the PF_6_^–^ septet is minimal. However, the effect on the degradation species is significant, and after 0.5 mM Mn(TFSI)_2_ is added to the electrolyte solution, all degradation species are undetectable. Although Ni(TFSI)_2_ causes a lesser degree of peak broadening, dramatic peak broadening and signal loss occurs in the sample containing 5 mM Ni(TFSI)_2_. These results are consistent with the ^19^F results observed in Figure 4, as the same fluorophosphate degradation species are being probed in Figures 4 and 5.

**Figure 6:**
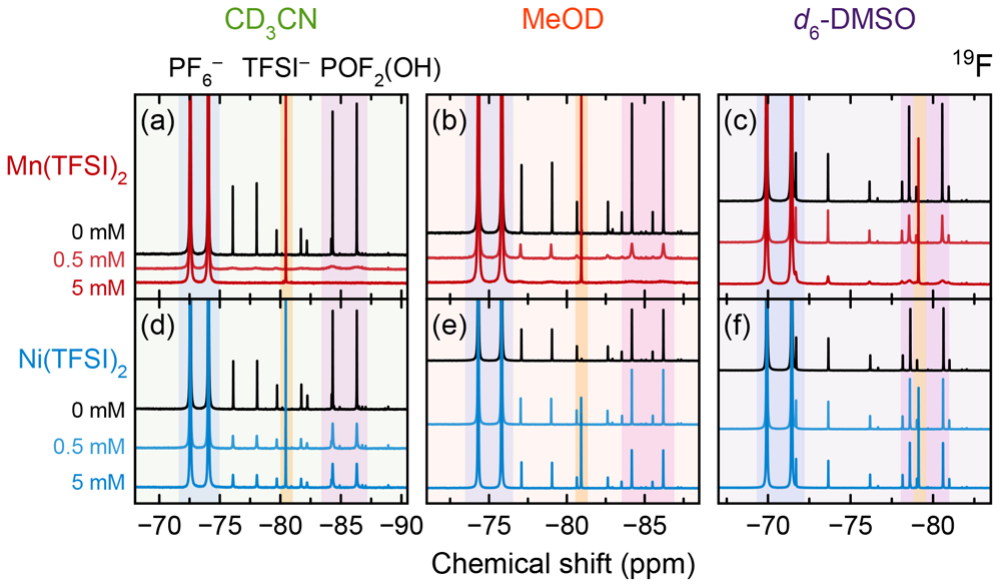
^19^F NMR spectra using (a, d) CD_3_CN, (b, e) MeOD, or (c, f) DMSO-*d*_6_ to dilute degraded electrolyte solutions containing Mn(TFSI)_2_ or Ni(TFSI)_2_. Solvents were mixed with electrolyte solutions in a 9:1 ratio (v/v). The concentrations of Mn(TFSI)_2_ or Ni(TFSI)_2_ listed indicate the concentration in the original electrolyte solutions; concentrations in the overall solutions are therefore 10% of these values. The singlet at −80.4 (CD_3_CN), −80.9 (MeOD), or −79.1 ppm (DMSO-*d*_6_) is due to TFSI^–^.

Figure 6 shows ^19^F NMR spectra for degraded electrolytes containing 0, 0.5, or 5.0 mM Mn(TFSI)_2_ or Ni(TFSI)_2_ that were diluted 10× with deuterated solvents. For solutions containing Mn(TFSI)_2_, surprisingly, the peak resolution varies by solvent: in acetonitrile, peak resolution is vastly reduced at 0.5 mM; in methanol, most peaks are still well-resolved at 0.5 mM but undetectable at 5 mM; and in DMSO-*d*_6_, all peaks can still be resolved at 0.5 mM and some peaks can still be resolved at 5 mM. When 0.5 mM Mn(TFSI)_2_ is diluted with deuterated solvents, the peak widths at half height of the two peaks in the POF_2_(OH) doublet are ∼63× larger in CD_3_CN (∼390–400 Hz vs 6.1–6.4 Hz), ∼11× larger in MeOD (∼47.8–48.4 Hz vs 4.4–4.5 Hz), or 3.4× larger in DMSO-*d*_6_ (16 Hz vs 4.6 Hz) than in the analogous diamagnetic CD_3_CN, MeOD, or DMSO-*d*_6_ solution. When 5 mM Mn(TFSI)_2_ is diluted with DMSO-*d*_6_, the peak width at half height of POF_2_(OH) is ∼31× larger (∼145 Hz vs 4.6 Hz). The peak width at half height of HF is not shown here, but for 0.5 mM Mn(TFSI)_2_, it is <2× larger in all solvents. For solutions containing Ni(TFSI)_2_, peaks can be resolved in all solvents at both Ni^2+^ concentrations. The peak positions also differ among the solvent systems, due to solvation effects.

**Figure 7:**
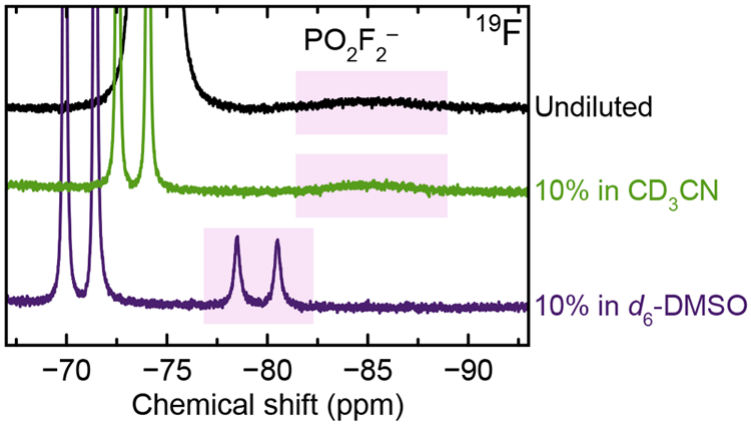
^19^F NMR of electrolyte that had been stored with LiMn_2_O_4_ powder at 60 °C for 11 weeks. Measurements were performed on the neat electrolyte and electrolyte that was diluted with CD_3_CN or DMSO-*d*_6_ (9:1 v/v). Pink shaded areas indicate PO_2_F_2_^–^. The sample not diluted with deuterated solvent (black curve) is referenced to PF_6_^–^ at −74.40 ppm to eliminate the bulk magnetic susceptibility shift.

Figure 7 shows ^19^F NMR spectra of an electrolyte solution that was stored with LiMn_2_O_4_ powder at 60 °C for 11 weeks. ICP-OES measurements indicated 4.67 ± 0.05 mM Mn was present in solution. NMR measurements were performed on the undiluted sample and on the sample after 10× dilution with deuterated acetonitrile or DMSO. The PO_2_F_2_^–^ signal (pink shaded area) appears as a broad unresolved peak in the undiluted sample and in the acetonitrile-diluted sample, whereas the signal appears as a well-resolved doublet in the DMSO-diluted sample.

**Figure 8:**
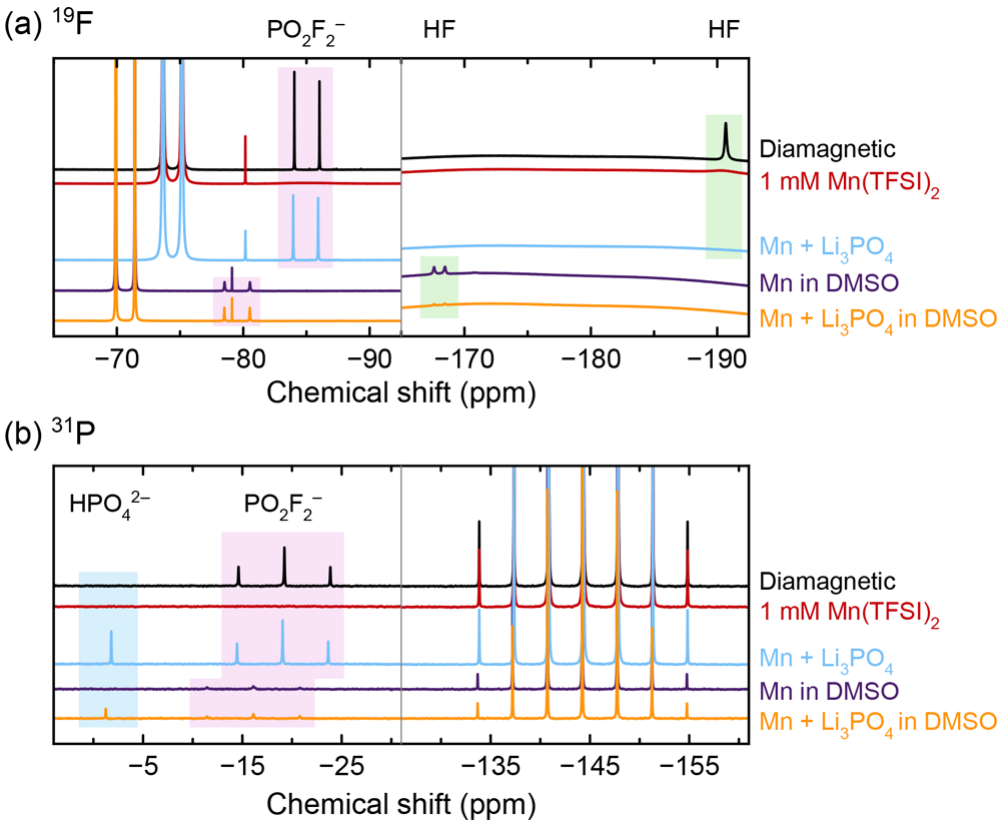
(a) ^19^F and (b) ^31^P NMR of degraded electrolyte solutions. Measurements were performed on the neat diamagnetic electrolyte solution, then rerun after the addition of 1 mM Mn(TFSI)_2_ and again after shaking with 10 mg Li_3_PO_4_. The Mn^2+^-containing electrolyte was diluted 9:1 with DMSO-*d*_6_, and this solution was also shaken with 10 mg Li_3_PO_4_. Pink shaded areas indicate PO_2_F_2_^–^; green shaded areas indicate HF; the blue shaded area is attributed to Li_2_HPO_4_/HPO_4_^2–^. The ^19^F singlet at −80.2 ppm (undiluted) or −79.1 ppm (DMSO-*d*_6_) is due to TFSI^–^. Samples not diluted with DMSO-*d*_6_ are referenced to PF_6_^–^ resonances at −74.40 ppm (^19^F) and −144.34 ppm (^31^P) to eliminate any bulk magnetic susceptibility shift.

Figure 8 shows ^19^F and ^31^P NMR spectra of a heat- and water-degraded electrolyte solution (not the same solution used in Figures 3–6) to which Mn^2+^, Li_3_PO_4_, and DMSO-*d*_6_ were added afterward, to examine strategies of mitigating paramagnetic spectral broadening. After the addition of 1 mM Mn(TFSI)_2_, the PO_2_F_2_^–^ signal (pink shaded area) is lost, and the HF signal (green shaded area) is greatly broadened. The paramagnetic solution was then subdivided, and some of it was shaken with Li_3_PO_4_ to precipitate the Mn^2+^.^137^ In this spectrum (light blue), the PO_2_F_2_^–^ signal is recovered, while the large HF signal is lost entirely; additionally, a new, small peak appears in the ^31^P NMR spectrum (blue shaded area; we attribute this to Li_2_HPO_4_ or HPO_4_^2–^). When the paramagnetic solution is diluted 10× with DMSO, the PO_2_F_2_^–^ signal is recovered and the HF signal becomes better resolved. When the paramagnetic solution is diluted 10× with DMSO and then shaken with Li_3_PO_4_, the PO_2_F_2_^–^ signal is recovered, the HF signal becomes significantly smaller, and a new ^31^P peak appears. The difference in the PO_2_F_2_^–^ signal between the sample diluted with DMSO and the sample that was diluted with DMSO and then shaken with Li_3_PO_4_ appears negligible. The ^19^F and ^31^P signals are also far smaller in the DMSO-diluted samples, due to having less electrolyte in the samples.

## Discussion

From the NMR spectra of pristine electrolyte solutions containing dissolved transition metals (Figures 1 and 2), it is apparent that large quantities of Mn^2+^ induce considerable broadening of the solvent and salt peaks, but Ni^2+^ does not; beyond this simple trend, it is also clear that there is considerable variation in how the different ions and molecules are affected by Mn^2+^. To evaluate these results further, the theory of paramagnetic relaxation is now briefly introduced. Applying Solomon–Bloembergen–Morgan theory to this system,^128−131,133^ the terms that affect the transverse relaxation rate of a nucleus coordinated to a paramagnetic ion, *R*_2M_, are shown in eq 1, where the first term is a dipolar term and the second term is a contact term. The dependence on spin (*S*(*S* + 1) terms) indicates that the relaxation rate increases as the number of unpaired electrons on the paramagnetic ion increases. Other symbols correspond to permeability of a vacuum (μ_0_), nuclear gyromagnetic ratio (γ_I_), electron spin g-factor (*g*_e_), Bohr magneton (μ_B_), distance between the nucleus and paramagnetic ion (*r*), and the correlation time for the dipolar term (τ_c_^dip^), Larmor frequencies for the nuclear spin (ω_I_) and electron spin (ω_S_), the hyperfine interaction constant (*A*/*ℏ*), and the correlation time for the contact term (τ_c_^con^).
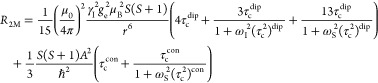
1The correlation time for the dipolar term is expressed in eq 2 as a function of the rotational correlation time τ_r_, electronic relaxation time τ_e_, and chemical exchange time between bound and unbound states τ_M_, where the shortest of these will be the dominant factor. The correlation time for the contact term is a function of only τ_e_ and τ_M_ (eq 3).
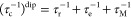
2
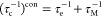
3

As the paramagnetic ion concentration increases, the nucleus under observation is on average located near more paramagnetic ions, and the difference between the observed *R*_2_ and the diamagnetic relaxation *R*_2d_ (i.e., the relaxation enhancement due to diamagnetic terms, such as the fluctuating fields caused by nuclear dipolar relaxation or the chemical shift anisotropy) is proportional to the paramagnetic metal concentration.^128^ This is shown in eq 4, where *f*_M_ is the molar fraction of nuclei that are bound to paramagnetic metal ions, which incorporates the metal concentration, the concentration of the species the metal is bound to, and the metal solvation number, and Δω_M_ is the hyperfine shift.^128,138^
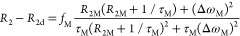
4

Lastly, in all solutions, the observed *R*_2_ is directly proportional to the spectral peak width at half-maximum, Δ*ν*_1/2_ (assuming other sources of line broadening can be ignored, e.g., from shimming), as shown in eq 5:^128^
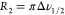
5

The increased broadening for Mn^2+^ is consistent with Mn^2+^ having a larger number of spins, and thus spin quantum number *S* (*S* = 5/2 for Mn^2+^ and *S* = 1 for Ni^2+^) and a much longer electronic relaxation time τ_e_ (τ_e_ ≈ 10^–8^ s for Mn^2+^ and τ_e_ ≈ 10^–10^ s for Ni^2+^).^128^ Mn^2+^ has a longer τ_e_ because it has an A (isotropic) spin state and has no spin–orbit coupling. Still, even at high Mn^2+^ concentrations, the main solvent and salt peaks are not broad enough to fall below the limit of detection (Figures 1 and 2). The solvent and salt peaks are also of the least concern because the experimentalist is likely to already know the identities of these species. However, degradation species are at risk of failing to be detected by NMR (Figures 3–5), particularly those in ^19^F NMR spectra. The ^19^F spectra show more loss of resolution than the ^31^P spectra: in pristine electrolytes, at 5 mM Mn(TFSI)_2_, the width at half height of the central peak in the ^31^P PF_6_^–^ septet is 3.9× broader than its diamagnetic analogue (33 Hz vs 8.5 Hz), whereas the widths at half height of the two peaks in the ^19^F PF_6_^–^ doublet are 20× broader than their diamagnetic analogues (102 Hz vs 5.0 Hz; Figure 2). The more severe ^19^F broadening is a consequence of the structure of PF_6_^–^, where outer F atoms shield the inner P atom from interaction with Mn^2+^, which reduces the distance-dependent dipolar relaxation enhancement (eq 1). The magnitude of the hyperfine interaction constant *A*/*ℏ* is also likely smaller for ^31^P than ^19^F for the same reason, and the ^31^P gyromagnetic ratio γ_I_ is smaller. The result of significant spectral broadening with dissolved Mn^2+^ has impacts for the NMR analysis of various cell chemistries, as dissolution from Mn-based cathodes also occurs in sodium-ion, potassium-ion, and zinc-ion cells.^139,140^

The broadening in the ^1^H spectra caused by the addition of transition metal salts to degraded electrolytes (Figure 3) appears relatively minor compared to the broadening in the ^19^F and ^31^P spectra (Figures 4 and 5). It is difficult to quantitatively compare the broadening among the nuclei, as the peaks in ^19^F and ^31^P spectra versus ^1^H spectra generally correspond to different degradation species. However, at only 0.01 mM Mn^2+^, for POF_2_(OH), the width at half height of the central peak in the ^31^P triplet is 3.3× broader than its diamagnetic analogue (105 vs 32 Hz), whereas the widths at half height of the two peaks in the ^19^F doublet are 5.9–6.2× broader than their diamagnetic analogues (43–45 Hz vs 6.9–7.6 Hz; Figures 4 and 5). By contrast, at 0.01 mM Mn^2+^, peak widths of any degradation species that could be quantified in the ^1^H spectra showed broadening of <2× (noting that many peaks were too small to be quantified and overlapped with other peaks, but this result can be qualitatively observed in Figure 3, expanded view). The ^1^H peaks at higher chemical shifts generally appear to undergo worse broadening/signal loss than peaks at lower chemical shifts, presumably because ^1^H nuclei that give rise to peaks at higher frequencies are generally closer to electron withdrawing groups (e.g., an O atom), and these groups may bind or interact more strongly with the paramagnetic ions. While the broadening of any given peak depends on the molar fraction of nuclei coordinated to the paramagnetic ion (*f*_M_), the broadening of each peak will further differ depending on several other variables in eqs 1–3, including the metal–nucleus distance, the hyperfine interaction constant, and the various correlation times. The discernment of peaks will also be worsened by overlapping resonances, where shoulder peaks are easily lost (Figure 3, 3.63 ppm expanded view). For ^1^H environments on the same molecule, the selective broadening of one environment over others may reveal the metal binding site. However, when comparing different molecules, increased broadening may be a result of a longer τ_c_^dip^, longer τ_c_^con^, shorter *r*, or larger *A*/*ℏ*, not necessarily a larger *f*_M_. It is therefore not trivial to determine, from analysis of the peak broadening, which degradation species are the most favored for Mn^2+^ coordination.

Similarly, the greater severity of ^19^F broadening (Figure 4) compared to ^1^H broadening (Figure 3) may result from a difference in *f*_M_, *r*, *A*/*ℏ*, and/or differences in correlation times. If Mn^2+^ coordinates more closely to F atoms in fluorophosphate degradation products than to H atoms in solvent degradation species, then it may seem that Mn^2+^ has a higher affinity overall for fluorophosphate species than for organic solvent degradation species. Equally, if an environment is simply farther from the Mn^2+^ binding site (with a larger *r* and smaller *A*/*ℏ*), it could appear that the entire molecule has a smaller Mn^2+^ affinity, when that may not be true. An example of this in the ^19^F spectra is the TFSI^–^ peak, which is narrow even with 5 mM Mn^2+^ in solution (Figure 4); this does not necessarily prove that Mn^2+^ does not coordinate to TFSI^–^, but rather that the −CF_3_ groups being probed by ^19^F NMR are not close to the TFSI^–^ Mn^2+^–O binding site.^141^ The differential relaxation enhancement may also result from differences in exchange rate, where *r* could be identical but a change in τ_M_ could dramatically change the relaxation rate; thus, peaks subject to less broadening may simply be undergoing Mn^2+^ exchange in the coordination shell at a different rate. The peaks from PF_6_^–^ degradation species,^91−93,96^ which tend to be similar in structure,^142−145^ undergo similar levels of broadening to one another; meanwhile, the PF_6_^–^ peak itself does not undergo significant broadening in degraded solutions. Preferential broadening of the PO_2_F_2_^–^ peak relative to the PF_6_^–^ peak has been previously observed and quantified, ascribed to preferential Mn coordination in a sample likely subject to transition metal dissolution.^99^ If Mn^2+^ coordination to PF_6_^–^ degradation species is indeed favorable, this may affect Mn dissolution from the cathode and its incorporation into the anode SEI. We further explore transition metal coordination in solution in upcoming work with targeted measurements of NMR relaxation rates and hyperfine interactions, as determined by EPR spectroscopy.

When electrolyte solutions are diluted with deuterated acetonitrile, methanol, or DMSO, the broadening of fluorophosphate degradation peaks varies (Figure 6). If the broad ^19^F signals are caused by Mn^2+^ coordination, the improved signal resolution with a given solvent suggests that the solvent can competitively coordinate Mn^2+^, disrupting coordination between Mn^2+^ and PF_6_^–^ degradation species (possibly decreasing the fraction of fluorophosphate species in the Mn^2+^ solvation shell). The results show the solvents’ ability to coordinate Mn^2+^ in degraded LiPF_6_ electrolytes follows the order DMSO > methanol > acetonitrile (Figure 6). This is consistent with the measured heat of transfer of Mn^2+^ from water to a second solvent (Δ_tr_*H*°), which is most negative (favorable) when the second solvent is DMSO and least negative (unfavorable) when the second solvent is acetonitrile,^146,147^ as well as with solution extended X-ray absorption fine structure (EXAFS) measurements showing that the DMSO Mn^2+^–O bond distance is shorter than the acetonitrile Mn^2+^–N bond distance.^147^ Broadening results in Figure 6 are also consistent with the calorimetrically determined donor numbers of 13.9–14.1 kcal·mol^–1^ for acetonitrile,^148,149^ 19.08 kcal·mol^–1^ for methanol^150^ (other methods yield ∼20–26 kcal·mol^–1^),^151−154^ and 29.8 kcal·mol^–1^ for DMSO.^148^ However, this solvent ranking is not consistent with the dielectric constants of 36.64 for acetonitrile, 33.0 for methanol, and 47.24 for DMSO.^155^ The donor number may therefore be more relevant than the dielectric constant for assessing Mn^2+^ solvation in these solutions, a conclusion also drawn for solvation/dissociation in various other contexts.^156−164^

A solvent’s ability to disrupt existing ion pairs in solution depends on the strength of the ion pair; hence, the NMR dilution results are likely applicable to other MPF_6_ electrolyte solutions, but may not be equally applicable to electrolytes that interact more strongly with Mn^2+^. Furthermore, because there may be different coordination environments in one solution (resulting from coordination to multiple degradation species), some peaks may see more broadening than other peaks even after *d*-solvent addition, as the transition metal complexes may have different hyperfine interactions and different coordinated fractions. The fluorophosphate peaks in Figure 6 appear to broaden similarly, but the HF peaks are not broadened significantly (perhaps due to the peak already being already extensively broadened by exchange processes in diamagnetic solution). Different solvent viscosities may also result in different rotational correlation times and exchange times, further contributing to variable peak broadening; additionally, motional processes at different temperatures will affect the degree of paramagnetic broadening.^128^ The broadening observed with all Ni^2+^-containing solutions is much smaller than that observed with Mn^2+^-containing solutions, so the choice of deuterated solvent is not likely to affect the analysis of degradation species generated in cells with Ni-based, Mn-free cathodes.

When Mn is added to the electrolyte via dissolution from LiMn_2_O_4_ powder (Figure 7), the findings remain the same as in the Mn(TFSI)_2_ samples, with the PO_2_F_2_^–^ signal best resolved in the sample diluted with DMSO. This supports the use of Mn(TFSI)_2_ as a model compound for Mn that is dissolved in situ from cathode materials and suggests that the findings in this work may be applied to electrolytes from cycled cells. While in recent years there has been discussion about whether some fraction of Mn dissolved from cathodes may exist as Mn^3+^,^23,48,52,62,63,121,123^ it is nonetheless generally agreed that some Mn^2+^ is present. Regardless, Mn^3+^ is paramagnetic (*d*^4^) and should also induce some degree of peak broadening.

Another route explored for minimizing the effect of paramagnetic ions on the ^19^F NMR spectra of electrolytes was direct precipitation of the metal using Li_3_PO_4_ (Figure 8), which has the benefit of not diluting the compounds of interest. The near-disappearance of the PO_2_F_2_^–^ doublet after the addition of Mn^2+^, and the signal’s subsequent recovery after the addition of Li_3_PO_4_, confirms that the Mn^2+^ was successfully removed from solution, presumably as a mixed Li and Mn compound, Li_3–*x*_Mn_*x*/2_PO_4_, or Mn_3_(PO_4_)_2_. However, the HF signal that is clearly resolved in the diamagnetic sample and observed as a broad peak in the Mn^2+^-containing sample was not recovered upon addition of Li_3_PO_4_. Reaction between HF and Li_3_PO_4_ likely caused F^–^ precipitation as LiF, possibly driven by the insolubility of LiF. This is supported by previous observation of lower levels of HF in LiPF_6_ solutions cycled in cells with Li_3_PO_4_-coated cathodes versus uncoated cathodes, where the authors proposed Reaction R1.^165^
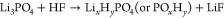
R1

The ^31^P NMR of the electrolyte following Li_3_PO_4_ addition shows a small singlet at −1.80 ppm (or −1.25 ppm in the DMSO-diluted sample). Published ^31^P shifts of H_3_PO_4_, H_2_PO_4_^–^, HPO_4_^2–^, and PO_4_^3–^ are similar to this, with more negative shifts as the phosphate ions are deprotonated.^166,167^ Addition of Li_3_PO_4_ to pristine electrolyte did not yield a new ^31^P singlet. Addition of Li_3_PO_4_ to diamagnetic HF-containing electrolyte produced a ^31^P singlet (and loss of the HF peak), confirming that the product results from reaction between Li_3_PO_4_ and HF. From relative peak widths (i.e., comparing the intensities of the HF peak that disappeared and the ^31^P singlet that appeared), there is a 10:1 ratio of HF consumption/H_*x*_PO_4_ generation, suggesting most of the H_*x*_PO_4_ reaction product is solid, likely Li_2_HPO_4_. This assignment is further supported by NMR analysis of reference compounds: aqueous H_3_PO_4_ and solid Na_2_HPO_4_ added to pristine electrolyte produced ^31^P signals at 0.84 ppm and −2.04 ppm, respectively. The −2.04 ppm of Na_2_HPO_4_ signal is close to the new −1.80 ppm ^31^P singlet. It is also possible that a small amount of Li_2_HPO_4_ reacts further to yield LiH_2_PO_4_: if a small amount of H_2_PO_4_^–^ is in exchange with HPO_4_^2–^, then that may explain why the new ^31^P shift is slightly downfield of the Na_2_HPO_4_ reference.

Thus, while Li_3_PO_4_ addition is a viable method to remove Mn^2+^ from NMR samples and recover fluorophosphate peaks, this method will result in the loss of HF peaks. The new ^31^P peak cannot necessarily be used to quantify the HF, because most of the Li_2_HPO_4_ is a precipitate. The negligible difference in the DMSO spectra with and without Li_3_PO_4_ suggests that DMSO coordinates Mn^2+^ sufficiently to recover NMR peaks without requiring Li_3_PO_4_ addition, although the ^19^F and ^31^P peaks are less intense in the DMSO-diluted sample than in the undiluted sample (due to the dilution). DMSO addition is suitable if there is a small sample volume available or the HF concentration is of interest. That said, loss of HF may sometimes be desirable if samples require storage in glass vessels, as this would prevent borosilicate etching.

The direct removal of Mn^2+^ has the further benefit of ensuring all chemical shifts are in the expected positions. Although we have largely ignored the chemical and hyperfine shifts in this work in favor of studying spectral broadening, we now return to the BMS shift.^128,135,168^ Magnetic susceptibility effects should not pose an issue in cases where the solvent is mixed directly with the electrolyte solution, as internal referencing corrects for the BMS. If the spectrum is referenced to a solvent that is physically separated from the paramagnetic electrolyte solution (e.g., a solvent capillary), the electrolyte components may be affected by the BMS shift, theoretically leading to shifts inconsistent with literature values. However, this effect is modest: theoretical peak shifts of 0.06 ppm for 1 mM Mn^2+^ and 0.01 ppm for 1 mM Ni^2+^ at 25 °C and 500 MHz are calculated using the spin-only moments,^128,135,169^ and experiments have shown BMS shifts of 0.06 and 0.02 ppm, respectively.^136^ In the case of Mn^2+^, the severe spectral broadening would make it clear that paramagnetic contaminants are present long before any issues arise with assigning peaks. Paramagnetic solutes also affect chemical shifts via hyperfine shifting, which includes contact and pseudocontact components.^128,129,170,171^ The various components of the electrolyte solution may coordinate to the paramagnetic solute differently, such that even a correctly referenced spectrum may yield some peaks that match literature values while others do not. As with the BMS shift, we do not expect this to cause significant undetected issues in most cases, as either the metal concentration should be too small to cause severe peak shifting, or the spectral broadening should make it clear that the solution contains paramagnetic contaminants.

A summary of the broadening mitigation methods is presented in Table 1. The dilution method was designed to be representative of samples extracted from coin cell parts, with the assumption that sample volume is limited or cannot be controlled; however, with sufficiently large samples, the DMSO dilution may perhaps be optimized to maximize peak narrowing while minimizing ^19^F and ^31^P signal reduction (e.g., 2:1 instead of 9:1 dilution). We also note that compounds other than Li_3_PO_4_ may be used to complex or chelate dissolved Mn^2+^ and reduce its effect on NMR spectra without causing HF consumption. The Mn-chelating abilities of various compounds (typically polymers) have been explored in electrolyte solutions with an aim toward reducing metal dissolution and deposition^123,172−180^ and may be suitable for use in mitigating peak broadening, as long as ^1^H signals from the chelating agent do not overlap with ^1^H signals of interest.

**Table 1:** Summary of Benefits and Drawbacks of the Two Methods Used in This Work to Counteract Paramagnetic Contamination of Electrolyte Solutions^a^

	Li_3_PO_4_ addition to pure electrolyte solution	DMSO dilution of electrolyte solution
electrolyte sample size	100s of μL	10s of μL
^31^P	more intense resonances	weaker resonances (due to smaller electrolyte volume in sample)
	new, small HPO_4_^2–^ signal*	
^19^F	more intense resonances	weaker resonances
	loss of HF peak*	
sample preparation	if using field locking, requires *d*-solvent capillary or coaxial NMR tube.	no modifications needed
	time required for added Li_3_PO_4_ to settle out of the sample*	
chemical shifts	no paramagnetic effects, but peaks should be referenced to shifts measured in electrolyte solutions	may be altered slightly by paramagnetic hyperfine shifts, but generally easier to find published shift references in DMSO
		potential sample contamination from impure DMSO (especially water)

a Asterisks (*) denote drawbacks to Li_3_PO_4_ addition that could be mitigated with the use of a different precipitation/chelation agent.

From this work, we now attempt to predict whether other battery-relevant dissolved metals may also pose an issue for NMR analysis. Generally, in light of the results from this work and literature values for transition metal electron spin relaxation times,^128^ it appears possible that dissolved Cu^2+^ (τ_e_ ≈ (1–5) × 10^–9^ s, *S* = 1/2), high-spin Fe^3+^ (τ_e_ ≈ 10^–9^–10^–10^ s, *S* = 5/2), and Mn^3+^ (τ_e_ ≈ 10^–10^–10^–11^ s, *S* = 2) may induce problematic broadening in NMR spectra of degraded electrolyte solutions, while high-spin six-coordinate Co^2+^ (τ_e_ ≈ 5 × 10^–12^–10^–13^ s, *S* = 3/2) and high-spin six-coordinate Fe^2+^ (τ_e_ ≈ 10^–12^–10^–13^ s, *S* = 2) are less likely to induce observable broadening. Dissolved Cu^+^, Al^3+^, and low spin Co^3+^ are diamagnetic and therefore will not induce any paramagnetic peak broadening. We suggest that samples with Cu^2+^ dissolution (from current collectors)^181^ or Fe^3+^ dissolution (from LiFePO_4_)^182^ should be treated with caution, although more work is needed to determine the severity of spectral broadening in nonaqueous battery electrolytes in such cases. Although Cr and Fe dissolution occur from steel battery casings,^35,183,184^ the oxidation states of these species are not yet clear. Lastly, while Mn^2+^-induced broadening is severe, we note that the electrolyte salt affects the rate of metal dissolution,^31,52^ so the use of a salt that causes less Mn^2+^ dissolution may also prevent these NMR analysis issues.

## Conclusions

The paramagnetic metals dissolved from lithium-ion battery cathodes can cause broadening in NMR spectra of electrolyte solutions, which can lead to difficulty identifying all degradation products. Broadening of resonances arising from major degradation products may indicate that other resonances from minor degradation products have been lost. The broadening caused by Ni^2+^ is not likely to be problematic, even in cases where high concentrations of dissolved Ni^2+^ ions are expected, e.g., in electrolytes from cells containing Ni-rich cathode materials. However, the broadening caused by Mn^2+^ can be severe even at low concentrations. In LiPF_6_ solutions, peaks from fluorophosphate species in ^19^F and ^31^P spectra are more likely to be affected than peaks from degradation species in ^1^H spectra. To mitigate this loss of signals from degradation species, dilution of the extracted electrolyte solution with high-donor number solvents is recommended, particularly DMSO-*d*_6_. For NMR of undiluted electrolytes, addition of Li_3_PO_4_ can remove Mn^2+^ from solution, but it also removes HF; other agents may be more suitable to precipitate or chelate paramagnetic contaminants and restore spectral resolution.
